# Impact of electronic prescribing on medication changes in users of multidose drug dispensing

**DOI:** 10.1016/j.rcsop.2025.100667

**Published:** 2025-10-02

**Authors:** Anette Vik Josendal, Trine Strand Bergmo

**Affiliations:** a PharmacoEpidemiology and Drug Safety Research Group, Department of Pharmacy, University of Oslo, Oslo, Norway; b Norwegian Centre for E-health Research, University Hospital of North Norway, Tromsø, Norway; c Department of Pharmacy, UiT, the Arctic University of Norway, 9037 Tromsø, Norway

**Keywords:** Electronic prescribing, Multidose drug dispensing, Workload, Drug treatment, Quality of health care, patient safety, Prescribing patterns

## Abstract

**Purpose:**

To investigate the number and type of prescription modifications after introducing e-prescribing for multidose drug dispensing (MDD) users.

**Methods:**

A longitudinal study using dispensing records from the main MDD supplier in Norway from June 2012 to August 2023. The study included 1522 MDD users with complete data from 24 weeks before and 24 weeks after the implementation. The main outcome measures were the number and type of prescription modifications.

**Results:**

In total, there was a 175 % increase in the frequency of prescription modifications, with 15.9 % of patients experiencing prescription alterations every two weeks, compared to 5.7 % before the intervention. Modifications were categorized into administrative and treatment changes. Administrative changes increased by 300 %, while treatment changes (including newly prescribed medications, discontinued medications, and dose adjustments) increased by 60 %. The proportion of patients with no prescription modifications throughout the 24 weeks decreased from 58.1 % to 26.2 % following the implementation of e-prescribing.

**Conclusion:**

Transitioning to an e-prescribing system is associated with more frequent modifications to patients' prescriptions. More frequent treatment changes can potentially improve medication safety and accuracy of the medication lists, but the major increase in administrative changes can also increase the workload of involved health care personnel.

## Highlights

• Prescription alterations increased with 175 % after e-prescribing was introduced.
• Administrative changes increased by 400 % and treatment changes by 60 %
• Both atients with alterations and mean number of alterations per patient increased.

## Introduction

The use of electronic prescribing has increased significantly in recent years. It is now widely used in the Nordic countries, Estonia, the Netherlands, the United States, Australia, and Canada.^1^^,^^2^ In Norway, e-prescribing was implemented in primary care in 2013, and today, over 95 % of the prescriptions are sent electronically (27 million per year).^3^ Prescriptions are transmitted directly from the prescribers to a central database, which stores the prescriptions until they are dispensed, expire, or a physician deletes them. All pharmacies have access to the central database, allowing the medicines to be dispensed from any pharmacy in the country. One of the few prescriptions still on paper is for individuals receiving prepackaged multidose dispensed drugs (MDD).

MDD is an adherence aid used by about 100,000 home-dwelling individuals who need help with their medicines, as well as residents in nursing homes.^4^ The medicines are machine-dispensed in unit-of-use disposable bags, with one bag for each dose occasion.^5^ The MDD system is implemented in primary care settings in the Nordic countries and the Netherlands.^6^^,^^7^ The rationale for using MDD in home care and nursing homes is that it reduces administration errors, reduce waste of unused medications and saves nursing time compared to filling dosette boxes.8., 9., 10.

Health authorities have started a national effort to include the prescriptions of MDD into the existing e-prescribing system. The initial pilot for the integration began in 2014, but the implementation has been slow due to technical challenges. At the time of data collection for this study, 984 general practitioners (GPs) had started using e-prescribing for about 6700 MDD-users.^11^ Under the previous paper-based system, the GPs printed medication lists and sent them via fax or secure messaging to the pharmacy, which then manually entered this information into the MDD system at the pharmacy. The paper medication list was valid for one year, provided there were no changes. The new electronic system allows GPs to enter the medications prescribed for MDD packaging in their electronic system and send them directly to the central database for e-prescriptions. The pharmacy responsible for MDD packaging is notified about new or updated prescriptions, which they can import directly into the MDD dispensing system. In the electronic system, every medication has its own prescription with an expiry date and a quantity prescribed. For more detailed information about the e-prescribing system and the pharmacists' control routines for MDD, see Jøsendal & Bergmo 2022.^12^

Previous research indicates that e-prescribing has the potential to improve physicians' workflow, increase pharmacy efficiency, and improve patient safety.^13^, ^14^, ^15^ However, studies have also found that e-prescribing introduces workarounds and alters work practices for the healthcare personnel involved.^14^^,^^16^^,^^17^ GPs using e-prescribing for MDD (eMDD) have reported that the system is less time-consuming and safer for the users compared to paper and fax.^18^^,^^19^ However, interviews with home care nurses and pharmacists have revealed that they experienced the system to be more time-consuming as they had to conduct more checks, both by reviewing the medication list when the GP made changes and all the extra individual e-prescriptions.^16^ Furthermore, a recent study found that the workload for pharmacists increased considerably after introducing eMDD due to an increased frequency of modifications to the prescriptions.^12^ After eMDD was implemented, 46 % of the prescriptions had modifications every other week, compared to 17 % before implementation, an increase of 170 %. This study solely focused on the total number of changes that required manual work from the MDD dispensing pharmacists and did not investigate the type of prescription modifications or the underlying reason for them. However, the authors proposed five potential mechanisms that might explain the rise in prescription modifications associated with the new system 1) fewer errors in the transmission of prescriptions, 2) the pharmacy gets notified about all changes from all physicians 3) more frequent renewing 4) mandatory sending of all prescriptions also those that are not dispensed as MDD and 5) changes in prescribing patterns. Considering that previous studies have shown that MDD users are prescribed more potentially inappropriate medications and have fewer changes in their medication treatments than patients with ordinary prescribing,^20^, ^21^, ^22^, ^23^ it is important to investigate these prescription modifications further. Therefore, we have initiated a study that aims to better understand these prescription modifications not just in terms of increased workload for pharmacies, but also to patient safety. The main objective is to retrospectively investigate the changes in prescription modifications over time, following the implementation of eMDD, by quantifying and characterizing the types of prescription modifications associated with the transition.

## Methods

### Study design and sample

We conducted a longitudinal study using anonymous prescription cards from the MDD supplier who first piloted the electronic prescribing system. They provided data from June 2013 (one year before the e-prescribing system was first piloted) to July 2023. A prescription card is a structured medication list that the MDD manufacturer uses to dispense MDD. This list contains all prescribed medications for a patient, including those not suitable for MDD and those taken *as needed*. We have only included the ones dispensed as MDD in this study, as this information corresponds 1:1 with dispensing records. The prescription card can include prescription drugs, over-the-counter medications, and dietary supplements if they are dispensed as MDD. Data about the medications/items dispensed include names, strength, formulation, ATC code,^24^ structured dosage schedule, prescribing physician, reimbursement codes and free-text instructions for use. Age and gender were given for the patients. The original dataset consisted of 6175 unique patients who had received MDD based on electronic prescriptions at least once during the study period.

### Analyses

MDD prescribing was transferred from paper to electronic prescribing continuously during the study period. This transition happened when the responsible prescribing physician got access to the electronic prescribing system. In our analyses, the date when the MDD for the first time was dispensed based on an electronic prescription was defined as the index date for each included MDD user.

**Inclusion Criteria:**

• MDD users who had complete data throughout the study period.
• Users who received MDD prescriptions both before and after the introduction of electronic prescribing.

**Exclusion Criteria:**

• Users who lacked complete data for all intervals (typically patients with hospital stays >14 days, patients transferred to nursing homes or who died during the study period).
• Users who did not have MDD before the introduction of electronic prescribing.

**Fig. 1:**
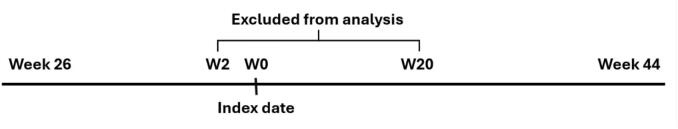
Time intervals included in the study. Index date (Week 0) = the first time the patient got multidose drug dispensing (MDD) based on electronic prescriptions.

In addition, we excluded 2 weeks prior to and 20 weeks after the index date (Fig. 1). This was based on the findings of another study, which showed an elevated rate of change in the weeks surrounding the transition, likely due to preparations for and adjustments to the new system.^12^ Since our study focus on the long-term effects of the new prescribing system rather than the transition phase, we excluded those weeks from our analyses.

Since most MDD is dispensed for 2 weeks at a time, we applied two-week intervals between each data point, covering a total of 24 weeks (6 months) before and 24 weeks (6 months) after the index date. We then had 12 data points before and 12 data points after the intervention. For each data point, we constructed a prescription list of current medications for each patient. The prescription list at each data point was compared to the next data point to classify the prescriptions as modified or not modified. We categorized modifications to prescriptions into two main groups:

• **Treatment changes:** constitute 1) discontinuation of medications, 2) newly prescribed medications and 3) dosage adjustments. The first two are defined by changes in the ATC code prescribed (active substance for dietary supplements), meaning that generic substitution would not be defined as a treatment change in this study if the dosage remained the same. Dose changes were defined as changes in the total daily dose of an ATC. The treatment changes were further categorized according to the ATC code of the changed medications.
• **Administrative modifications (other Changes):** All other changes to the fields on the pharmacy prescription card. This encompassed adjustments in reimbursement information, modifications in the free-text field used for indication and use of medications, and changes in the prescribing physician. Although these fields do not affect the medication being dispensed, changes in these fields necessitate a manual check by the pharmacist before dispensing.^12^

The classification of changes was discussed and defined by both authors in collaboration, with the first author and a software developer writing the code to implement it. We used Python 3.8 to compare prescription lists, and Stata (MP 15) and descriptive statistics to analyse the data. We used paired *t*-tests to compare the mean number of modifications before and after the transition.

### Ethics

This study was approved by the Data Protection Officer at the University Hospital of North Norway (UNN) (Project No. 02003). The MDD supplier who provided the data (Apotek 1 AS) anonymised the data before it was handed to the researchers. We did not need approval from the Regional Ethics Committee since it was a retrospective study, defined as health service research.

## Results

The original dataset consisted of 6175 patients who had received MDD based on electronic prescriptions at least once during the 10-year study period. We excluded 4653 patients who did not have continuous data for the entire period, which resulted in a total of 1522 users in the final analysis.

From Table 1, we find that the average age of included patients was 76 years, and an average of 5.8 (SD = 2.9) dispensed medications in their MDD bags. More women than men were included, and the women were older and used slightly more medicines than men. See Table 1.

**Table 1:** Sex, age, and number of medications in mean (SD) unless otherwise reported.

	Female	Male	Total
Sex	882 (58 %)	640 (42 %)	1522 (100 %)
Age	79.5 (17.5)	71.9 (18.9)	76.3 (18.5)
No. of drugs	6.1 (2.9)	5.4 (2.8)	5.8 (2.9)

### Patients with prescription modifications

**Fig. 2:**
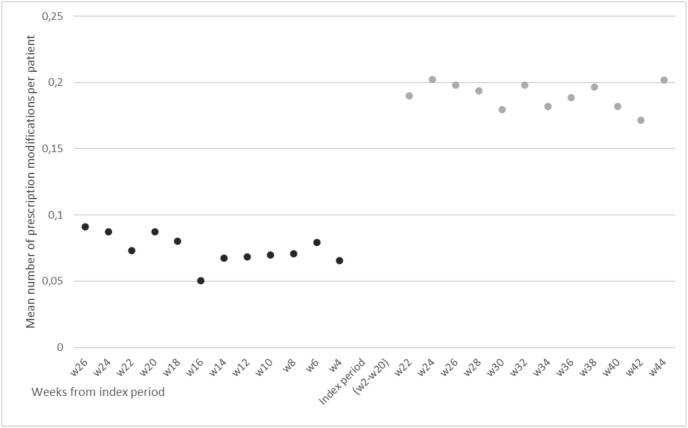
Mean number of prescription modifications for 1522 patients at two-week intervals, 24 weeks before and 24 weeks after the transition to e-prescribing.

As shown in Fig. 2 and Table 2, there is a significant increase in the mean number of prescription modifications after the transition to e-prescribing. The average number of prescription modifications per patient increased from 0.074 (*SD* = 0.114) to 0.190 (SD = 0.178), *t*(1521) = 21.23, *p* < .001. The number of increased modifications is relatively stable long term (Fig. 2).

**Table 2:** Prescription modifications before and after the e-prescribing intervention (N = 1522).

	Before (24 weeks)	After (24 weeks)	Difference
	Mean (SD)	Mean (SD)	Mean (SD)
Prescription modifications per patient	0.074 (0.114)	0.190 (0.200)	0.116 (0.213)⁎
	Number (%)	Number (%)	Number (%)
Average number of patients with prescription change every second week	87/1522 (5,7)	239/1522 (15,7)	152/1522 (10.0)
	Number (%)	Number (%)	Number (%)
Patients without any prescription changes during the entire 24-week period	884/1522 (58,1)	398/1522 (26,2)	486/1522 (32.0)

⁎ Significant difference (*P*-values ≤ .001).

The increase in the average number of prescription modifications is due to both an increased frequency of modifications as individual patients receive more frequent modifications after the implementation, but also an increased number of patients who have modifications. Looking at the entire 6-month study period before the intervention, over half of the patients (58.1 %) had no changes in their prescribing, while during the 6 months after the intervention, this decreased to 398 (26.2 %).

As shown in Table 2 we also find that the frequency of changes increase from an average of 5.7 % (87/1522) of patients having modifications in their medication list every second week in the period before the implementation, compared to 15.9 % (239/1522) after – a total increase of 175 %.

### Types of prescription modifications

**Fig. 3:**
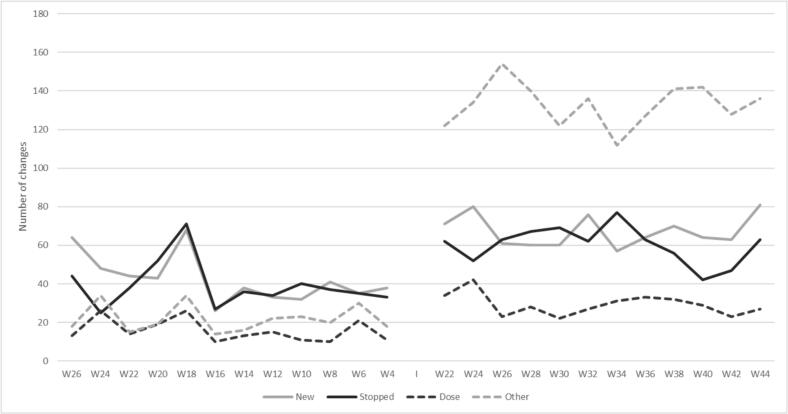
Number of prescription modifications categorized as new prescriptions, discontinued prescriptions, dose changes and other changes, before and after the implementation of the electronic prescribing system for multidose drug dispensing.

As shown in Fig. 3, all types of prescription modifications increased after the intervention; newly prescribed medications, stopped medications, dose changes and other changes, with the largest increase in the latter category. “Other changes”, which encompass adjustments in reimbursement information, modifications in free-text field used for indication and use of medications, and changes in the prescribing physician; increased from an average of 32 “other” modifications every 2 weeks to 132 – a 4-fold increase. For the treatment changes (newly prescribed, stopped and dose changes), these increased from an average of 98 changes per 2-week period to 157.

The different ATC codes involved in the treatment changes (newly prescribed, stopped medications and dose changes) are shown in Table 3. In absolute numbers, there is an increase in changes for all ATC codes from before to after the implementation, with the largest increase for medications in ATC group N (Nervous system).

**Table 3:** Treatment changes by ATC codes before and after the transition to e-prescribing.

ATC code	BEFORE		AFTER	
	Number of changes (% of total number of changes)		Number of changes (% of total number of changes)	
A- Alimentary tract and metabolism	206	(17.6 %)	372	(19.3 %)
B- Blood and blood forming organs	148	(12.6 %)	197	(10.2 %)
C- Cardiovascular system	241	(20.6 %)	396	(20.6 %)
G- Genito urinary system and sex hormones	26	(2.2 %)	45	(2.3 %)
H- Systemic hormonal preparations, exc. Sex hormones and insulins	27	(2.3 %)	39	(2.0 %)
J- Antiinfectives for systemic use	26	(2.2 %)	29	(1.5 %)
M- Musculo-skeletal system	26	(2.2 %)	44	(2.3 %)
N- Nervous system	346	(29.5 %)	656	(34.0 %)
R- Respiratory system	50	(4.3 %)	66	(3.4 %)
S- sensory organs	2	(0.2 %)	0	(0.0 %)
L- Antineoplastic and immunomodulating agents	0	(0.0 %)	0	(0.0 %)
V- various	0	(0.0 %)	1	(0.1 %)
Dietary supplements	73	(6.2 %)	83	(4.3 %)
SUM changes	1171	(100 %)	1927	(100 %)

## Discussion

This is one of the first studies looking at how electronic prescribing of MDDs affects prescribing patterns and drug treatment changes. This study shows that there is an association between the transition to electronic prescribing of MDD and an increased number of prescription modifications. After the transition, there is an increase both in the mean number of prescription modifications per patient and the number of patients with modifications. The increase is largest for administrative changes, but there is also a 60 % increase in treatment changes. Our study supports the claims of home care nurses and pharmacists using the new system, who describe the new system as safer, but also that it increases their workload.^16^

### Treatment changes and patient safety

Our study shows a significant increase in the frequency of drug treatment changes after transitioning to the electronic prescribing system. Fewer errors in the transmission and transcribing of prescriptions can be an important reason for this increase. In the paper-based system, there are many manual routines for updating, sending and transcribing prescriptions.^25^, ^26^, ^27^ Treatment changes from the GP might thus never get sent or not be registered correctly at the pharmacy. With the new system, many of these routines are automated, resulting in more treatment changes being timely and correctly implemented in the patient's MDD bags, also resulting in more correct and updated medication lists.^28^ Our study may also provide a mechanism for understanding how the e-prescribing system contributes to the reduction of discrepancies in medication lists shown in a previous study.^28^ By ensuring that changes in treatment are communicated accurately between healthcare providers, the electronic system can help to minimize discrepancies. This improved communication not only enables faster updates to treatment regimens but also aligns with the increased number of prescription changes observed in our study. Therefore, our findings contribute to the existing literature that e-prescribing has the potential to reduce medication discrepancies and improve patient safety by facilitating more timely treatment changes.

Another mechanism that might explain the increase in the number of changes is that the system now notifies the pharmacy about all changes, something that was not the case in the paper-based system.^12^ Previously, short-term treatments would often not be dispensed in MDD, and the GP could thus choose not to notify the pharmacy about these prescriptions. In the new system, however, the pharmacy will be notified about all changes to a patient's treatment. The increase in the number of discontinuations and new medications we find in our study might therefore be a result of more short-term treatments being dispensed for MDD. Looking at Table 2, we see that the relative number of changes related to ATC group N – Nervous system seems to increase after the implementation. Many of these medications, such as hypnotics and analgesics, are typically to be used only short term, and this might explain why there is an increase in changes for these medications. More frequent adjustments to medications like analgesics and hypnotics, which carry a risk of abuse, may also suggest more appropriate management of these drugs.

It has previously been shown that patients with MDD are prescribed more potentially inappropriate medications than patients with ordinary prescribing.^23^^,^^29^, ^30^, ^31^, ^32^ One suggested reason for this has been too automatic systems for renewing prescriptions in the MDD system, leading to physicians reviewing the medication treatment less frequently.^20^^,^^21^ In the paper-based system, GPs only needed to renew the prescription once a year, as the MDD prescription covered all medications for that duration. In contrast, the electronic system generates individual prescriptions for each medication, which may not last a full year. This requires GPs to review the medication list more frequently for renewal. While interviews with healthcare personnel using the eMDD system in Norway indicate that prescriptions are being renewed more often than in the paper-based system,^16^ this increased frequency of review may provide GPs with more opportunities to make necessary adjustments beyond mere renewals. This is one possible explanation for why we in our study find that nearly 60 % of patients had no changes to their prescriptions during the six months of the paper-based prescribing, which decreased to approximately one-fourth after the transition to electronic prescribing.

The increased number of treatment changes we find in our study also aligns with previous research indicating that the new system facilitates quicker adjustments to medication treatments delivered in MDD bags.^12^ As limited flexibility in adjusting medications and dosages has been a critique of the MDD system,33., 34, 35our findings indicate that the e-prescribing system might help to address this issue. In summary, while our study suggests that increased treatment changes can enhance patient safety through timely adjustments, it is important to recognize that despite the new system reducing certain errors—particularly those related to the inaccurate transfer of information—new types of errors may arise. Not all changes to medication treatments are necessarily appropriate for the patient, particularly when individual modifications are made without considering the treatment plan as a whole. This concern has been echoed in previous studies, which highlight that the increased complexity of the prescribing process, along with the involvement of multiple stakeholders, necessitates a clearer division of responsibilities.^16^^,^^28^ Such complexity can elevate workloads for healthcare providers, leading to greater miscommunication and an increased risk of errors. Consequently, further research is needed to explore these challenges and to ensure that patient safety and prescribing quality are prioritized in the devolopment of future healthcare systems.

### Administrative changes and increased workload

The largest increase in modifications we find in our study is in the category of “other” changes, with these being four times as common in the electronic system as the paper-based. The “other” category of changes includes changes in the free text field. This field is typically used to write the indication for use and information about generic substitution, neither of which one would expect to change more frequently with an electronic prescribing system. In addition, the “other category” includes changes in reimbursement information and changes in the prescribing physician. In the paper-based system, most changes would go via the GP, so there will only be one prescriber. However, in the electronic system, the pharmacy has direct access to prescriptions from the hospital and can use these to dispense MDD, but later adjustments or renewal of these prescriptions will mostly be done by the GP. One would thus expect more frequent changes in prescribing physicians with the new system. Similarly, although the reimbursement code is linked to diagnosis, which one would not expect to change frequently, a different classification system is used in primary (International Classification for Primary Care (ICPC-2)) and secondary care (International Classification of Diseases (ICD-10)). If a prescription is issued by a hospital doctor and is later renewed by a GP, this may result in the reimbursement code being changed as well. Although both prescribing physicians and reimbursement information are expected to increase in the new system, it seems unlikely that this would explain the entire four-fold increase we find in this study. These “other” changes should thus be looked at in more detail in further studies, to rule out technical errors, such as e.g. computer-generated text in the free text fields when prescriptions are renewed.

The studies that have shown MDD users to have fewer changes in their medication regimens than patients with ordinary prescribing^20^^,^^21^ have been done on electronic prescribing systems, but on systems where the prescribing procedure differs between ordinary and MDD prescribing. In our study, we find a three-fold increase in the number of prescription modifications per two-week period when transitioning from a system where the prescribing procedure differs between MDD (paper prescribing) and ordinary e-prescribing, to one where the prescribing procedures are the same. Our findings indicate that it may not be the MDD system in itself that leads to fewer changes in prescribing, but rather having different procedures for prescribing MDD and ordinary prescriptions. This is also supported by a Finnish study, where they have common prescribing systems for both prescription types.^36^ In our study, we also find that there is an increase in all types of changes and all ATC groups, indicating that the increase is likely due to the system, rather than behavior change from the prescribing physician.

Unlike the previous study on the electronic MDD system, we did not define the renewing of prescriptions as a prescription modification.^12^ While more frequent renewals have been suggested as a primary reason for the increased workload observed with the electronic prescribing system,^12^^,^^16^ our findings indicate that the increase in administrative changes encompasses much more than just renewals. Moreover, the significant increase in administrative prescription changes identified in our study suggests that the new system likely adds to the overall burden for healthcare providers, with the tasks related to renewing prescriptions contributing to this existing administrative pressure. In light of these findings, there is a pressing need for further research to investigate the underlying causes of the observed administrative changes and their effects on healthcare providers' workloads. Improvements, including a clearer delineation of responsibilities, enhanced communication pathways, and an optimized user interface design, could reduce the administrative burden on healthcare professionals and help the e-prescribing system achieve its goal of increasing medication safety.

### Strengths and weaknesses

The use of a complete data set for many patients over an extended period is the main strength of this study. It allows for a more comprehensive understanding of the trends and patterns in medication use and management among older adults with polypharmacy. The large sample size and longitudinal design increase the validity and generalizability of the findings. However, the lack of a control group is a notable weakness of the study. Without a control group, it is challenging to establish a causal relationship between the implementation of electronic prescribing for MDD users and the observed trends in prescription changes. While the longitudinal data provides valuable insights into the changes over time, a control group would have allowed for a more robust comparison and analysis of the impact of the electronic prescribing system. In addition, the study relies solely on medication lists without clinical data on the patients; this may impact the generalizability of the findings to broader patient populations and clinical contexts.

## Conclusion

Our study demonstrates that transitioning to an e-prescribing system is associated with an increased frequency of modifications to patients' prescriptions, both administrative changes and changes to medication treatment. These findings can be used to evaluate and inform the further implementation and adaptation of the e-prescribing system, ensuring that it meets the needs of healthcare providers and enhances patient safety. The increased frequency of treatment changes is likely to enhance medication safety and result in more accurate and up-to-date medication lists. However, further research is needed to examine the volume of administrative changes being made to the prescriptions, as these potentially increase workload without increasing patient safety. Additionally, future studies should investigate whether the new electronic system affects prescribing patterns and overall prescribing quality.

## CRediT authorship contribution statement

**Anette Vik Josendal:** Writing – review & editing, Writing – original draft, Methodology, Formal analysis, Data curation, Conceptualization. **Trine Strand Bergmo:** Writing – review & editing, Project administration, Methodology, Conceptualization.

## Declaration of competing interest

The authors declare that they have no known competing financial interests or personal relationships that could have appeared to influence the work reported in this paper.

## Glossary

MDD: Multidose drug dispening. Machine-dispensed tablets and capsules in unit-of-use disposable bags, with one bag for each dose occasion.
